# Supplementary material for: Estimation of Serial Interval and Reproduction Number to Quantify the Transmissibility of SARS-CoV-2 Omicron Variant in South Korea
Source: Viruses. 2022 Mar 4;14(3):533. doi: 10.3390/v14030533 (PMC8948735; doi:10.3390/v14030533)
Supplement: Supplementary file 1 [file viruses-14-00533-s001.zip › viruses-1599182-supplementary.pdf]

Table S1. Estimation of serial interval distributions, evaluated by fitting four different parametric distributions.

	**Normal**	**Log-normal**	**gamma**	**Weibull**
Mean (Days)	3.78	3.97	3.78	3.74
Standard deviation	3.33	12.98	6.65	1.00
AIC†	386.7	412.88	396.11	389.78

†Akaike information criterion.
